# Genetic diversity in *ex situ* populations of the endangered *Leontopithecus chrysomelas* and implications for its conservation

**DOI:** 10.1371/journal.pone.0288097

**Published:** 2023-08-02

**Authors:** Gabriela Guadalupe Aliaga-Samanez, Nathalia Bulhões Javarotti, Gisele Orecife, Karla Chávez-Congrains, Alcides Pissinatti, Cauê Monticelli, Mara Cristina Marques, Peter Galbusera, Pedro Manoel Galetti, Patrícia Domingues de Freitas

**Affiliations:** 1 Departamento de Genética e Evolução, Universidade Federal de São Carlos, São Carlos, São Paulo, Brazil; 2 Centro de Primatologia do Rio de Janeiro, Guapimirim, Rio de Janeiro, Brazil; 3 Departamento de Conservação e Pesquisas Aplicadas, Coordenadoria de Fauna Silvestre, Secretaria de Meio Ambiente, Infraestrutura e Logística do Estado de São Paulo, São Paulo, São Paulo, Brazil; 4 Zoológico de São Paulo (FPZSP), São Paulo, São Paulo, Brazil; 5 Antwerp Zoo Centre for Research and Conservation, Royal Zoological Society of Antwerp, Antwerp, Belgium; University of Veterinary Medicine Vienna: Veterinarmedizinische Universitat Wien, AUSTRIA

## Abstract

*Leontopithecus chrysomelas*, the Golden-headed Lion Tamarin (GHLT), is an endangered and endemic Neotropical primate from the Atlantic Forest of Brazil that has suffered a reduction of its habitat and population size in the wild. *Ex situ* populations have been established as a relevant alternative to safeguard the species and retain its genetic diversity and evolutionary potential. This study evaluated the genetic diversity and structure of the two main Brazilian captive populations of GHLT, which have been under human care at the Primatology Center of Rio de Janeiro (CPRJ) and the Zoological Park Foundation of São Paulo (FPZSP). Our results revealed levels of genetic diversity overall comparable to those observed for other *Leontopithecus* species and for *ex situ* and *in situ* populations of GHLT previously studied. Bayesian and principal coordinate analyses showed a moderate differentiation between CPRJ and FPZSP populations. Both populations presented observed heterozygosity values higher than expected heterozygosity values for most of the microsatellites used in this study, suggesting that the management has been efficient in avoiding an increase in homozygosity. However, simulations point to a significant loss of genetic diversity in the next 100 years, mainly in the FPZSP population. Such data are relevant for further decision-making on the metapopulation management of *L*. *chrysomelas* in captive conditions and for integrating *in situ* and *ex situ* conservation plans.

## Introduction

There is no longer any doubt that *ex situ* conservation is an insurance policy for endangered species worldwide. Reproducing and growing threatened organisms under human care, outside of their natural habitats, have been used for obtaining new individuals and restoring depauperate wild populations [1–3]. Nevertheless, captive breeding programs have important challenges for the maintenance of healthy populations [4]. The loss of genetic diversity, inbreeding and adaptation to captivity are virtually inevitable but should be minimized for the genetic health of the *ex situ* populations [5]. These effects are difficult to predict, given the wide range of life history characteristics of species (e.g. reproductive strategies) [5, 6]. Several primate species have captive populations included in national or international conservation programs [7–9], however, the genetic health of these populations is little known.

The Golden-headed Lion Tamarin (GHLT), *Leontopithecus chrysomelas* (Kuhl, 1820), is a Neotropical primate endemic to the Brazilian Atlantic Forest [10] and originally distributed in southeast Bahia and northeast of Minas Gerais states [11]. This species mainly uses lowland seasonal Atlantic rainforest, but also occupies secondary forest and *cabrucas*, which are areas destined to cacao agriforest plantations [11].

Despite the relatively high ecological plasticity of *L*. *chrysomelas*, its population size in the wild has decreased more than 60% in the last decades, mainly due to the continuous deforestation and subsequent habitat fragmentation and loss [12–14]. In addition, illegal activities, such as hunting, trading, and trafficking, have also negatively impacted the species [15]. Consequently, nowadays, the whole population of *L*. *chrysomelas* in nature is estimated to include approximately 2,500 mature individuals, distributed over a very restricted geographic area in southeast Bahia [16]. Thus, the species is currently listed as Endangered by the Red List of the International Union for Conservation of Nature [17]. A prior genetic analysis in wild *L*. *chrysomelas* [18] already revealed population structuring, possibly resulting from habitat fragmentation, and low levels of genetic diversity, which were similar to those of the other endangered *Leontopithecus* species [17, 19].

Efforts for the conservation of *L*. *chrysomelas* have been employed since the 1970’s, and include actions to protect populations in natural habitat, but also to establish viable *ex situ* populations as a manner to minimize the extinction risk [5, 19]. A review on the history of the captive program of *L*. *chrysomelas* mentions that the first *ex situ* colony of the species was established at the Tijuana Biological Park in Rio de Janeiro, Brazil, being later transferred to the Primatology Center of Rio de Janeiro [15]. In 1979 there were about 20 individuals in captivity in Brazil [20]. However, in the 1980’s some illegally exported animals were returned to the country, and a worldwide captive breeding program, along with the Studbook for the species, was initiated, hugely expanding the *ex situ* population size [21].

The GHLT captive breeding program showed a remarkable success over the years, evidencing rapid growth from 285 individuals kept in 22 institutions in 1989 to 611 ones distributed in 99 institutions at the end of 1999 [20]. In 2017 the Studbook of the species recorded 443 *L*. *chrysomelas* kept in 105 institutions in Europe, North America, Asia, and Brazil, including 22 countries [22]. In May 2022 there were approximately 393 extant *ex situ* GHLTs, 262 maintained overseas and 131 in 19 Brazilian institutions, of which the Zoological Park Foundation of São Paulo (FPZSP) and the Primatology Center of Rio de Janeiro (CPRJ) maintain the largest populations (Pissinatti A. and Monticelle C., personal communication). Currently, the species is distributed in 17 Brazilian institutions (S1 Fig), with FPZSP and CPRJ keeping, respectively, 18 and 45 animals under human care (Marques M.C., personal communication).

The current pedigree-based genetic diversity of the total Brazilian captive GHLT population is high (95%), due to a relatively large founder basis (43 founders). However, the pedigree of this population is known for only 73%, impairing an optimal genetic analysis of the breeding program [22]. In addition, pedigree-based analyses pointed to a decrease in their genetic diversity in the last 10 years [22]. Nevertheless, there is no molecular genetic information of these Brazilian captive populations thus far. For *ex situ L*. *chrysomelas*, molecular genetic assessments have been performed to date only in the population under human care in Europe [22], which showed low levels of genetic diversity, such as the Brazilian and European *ex situ* groups of the congeneric species *Leontopithecus chrysopygus*, that also demonstrated genetic differentiation in addition to accumulated inbreeding based on pedigree analyses [23, 24].

In this study, using a microsatellite panel previously employed for captive and wild populations of the focal species [18, 19], we characterized genetic diversity, genetic structure and inbreeding for *ex situ* populations of *L*. *chrysomelas* managed by the two main institutions that reproduce the species in Brazil for conservation proposes. Predictive analyses were also performed to simulate scenarios of genetic diversity reductions over generations, considering a retention of 90% of the current genetic diversity as a threshold for long term population viability [6, 25].

We hypothesized that the Brazilian captive populations show low genetic diversity and genetic differentiation among them. We also hypothesized that these populations will lose more than 10% of their current genetic diversity over the next 100 years, despite eventual influx of confiscated animals and exchange of individuals among institutions. We raised such hypotheses considering (i) the difficulty in making optimal breeding recommendations, due to the lack of genealogical information for 27% of the Brazilian captive GHLTs; and (ii) the decrease in their genetic diversity, detected by pedigree analyses [22]. Our findings raised relevant information for the Brazilian *ex situ* GHLT populations and for further conservation plans that consider molecular data for management practices.

## Material and methods

### Legal permits

This study was conducted according all legal and ethical standards and requirements established by the Biodiversity Authorization and Information System (SISBIO, MMA, Federal Government, Brazil, numbers 53201–1; 63477–3), and by the Ethics Committee in Animal Use and Experimentation (CEUA, Federal University of São Carlos, Brazil, number 7058110316). The access to genetic patrimony was registered at National Genetic Heritage Management System (SISGEN, MMA, Federal Government, Brazil, number A411359). Sample collections were carried out following the recommendations proposed for non-human primates by the American Society of Primatologists (ASP).

### Biological sampling and DNA extractions

We collected hair or blood samples from a total of 104 GHLTs, of which 55 were under human care at the Primatology Center of Rio de Janeiro (CPRJ); and 49 at the Zoological Park Foundation of São Paulo (FPZSP). This sampling included all extant adult individuals, inclusive the related ones, maintained in both institutions in 2014, when biological samples were collected (S1 Table). For blood collections, anesthesia procedures were first performed by induction with 2 to 5% isoflurane with oxygen at 2 L/min, using an inhalation equipment. Then, approximately 200 μL of whole blood from the femoral vein was collected, using vacutainers with EDTA (3.6 mg), and posteriorly kept at -20° C. We also manually collected tufts of hairs containing cell bulbs from the back of each animal and stored them individually in plastic envelops kept at room temperature.

After sampling, each animal was safely released in its respective enclosure. DNA extractions from blood and hair samples were carried out using prior protocols described, respectively, by Aljanabi & Martinez [26] and Sambrook *et al*. [27]. DNA quantity and quality was evaluated using a Nanodrop spectrophotometer (NanoVue Plus, GE Healthcare, Chicago, USA).

### Microsatellite amplification and genotyping

Eleven microsatellite loci were amplified, using seven species-specific primer pairs [19], and four heterologous primer pairs isolated for *L*. *chrysopygus* [28], as shown in Table 1. To select these loci, we first obtained information from previous studies that had already used these primer pairs in *L*. *chrysomelas* (18) and in congeneric species [28]. Then, we selected the loci that presented the best amplification patterns in our sampling.

**Table 1:** Information on successfully amplified microsatellite loci used in this study with primer sets previously described for *Leontopithecus chrysomelas* and *Leontopithecus chrysopygus*.

Species	Locus	Primers sequence (5’-3’)	Repeat	Fluorophore	T°C
***L***. ***chrysomelas***	*Lchu1*	F: GCTCAGGTGTTATTTATGTCCAAA	Tetra	PET	58°C
		R: GTTTCTTGCAACTATCTTGCATGTTCTGC			
	*Lchu3*	F: AAGGCATGATGTATCTTGTTCTCA	Tetra	FAM	58°C
		R: GTTTCTTATCTTTCTGTATGTGTCTCCCTGTCT			
	*Lchu4*	F: TGACCAAAGAAAATGCAAAA	Tetra	VIC	55°C
		R: GTTTCTTGCACAGGGTATTTAGCAGGA			
	*Lchu5*	F: TGATGCTAAAACAGAAGCATTT	Tetra	NED	55°C
		R: GTTTCTTGTCCTGATGTTCACAAAACCT			
	*Lchu6*	F: GCCTTAATTAGCACCAGAACC	Di	PET	55°C
		R: GTTTCTTACCACTCCAAGCCTTCAGTA			
	*Lchu8*	F: CACGGCAATGTGGGAATAA	Di	NED	58°C
		R: GTTTCTTTTCAGTAGTTGGGACTGGGATAA			
	*Lchu9*	F: TTCATTGTAGCATTGTTGGTCAT	Di	VIC	58°C
***L***. ***chrysopygus***	*Leon2*	R: GTTTCTTTTGCCTCCTCATAGTTCCTCAT			
		F: CTGCTTCTTGTTCCACTTCTTCTC	Di	FAM	56°C
		R: GTTTGGGTGGTTGCCAAG			
	*Leon21*	F: CAGTTGAGGGAACAGGAATTA	Di	PET	60°C
		R: CACTGCACTGACAGAGCAAG			
	*Leon27*	F: AAGCGCAGATTTATTGATAGG	Di	VIC	60°C
		R: TGCAGGTAAATGATGGTAATG			
	*Leon30*	F: GGACCTGATTGAAGCAGTC	Di	NED	60°C
		R: TTCCCTGAGAATCTAATGGAG			

F: forward, R: reverse, Tetra: tetraploid, Di: diploid, T°C: alignment temperature.

Polymerase Chain Reactions (PCRs) contained approximately 10 ng/μL of DNA obtained from blood or hair, 1x GoTaq Promega (Madison, Wisconsin, USA), 1x Buffer, 0.46 pmol of the reverse and M13 primers, 0.12 pmol of the forward primer, 0.30 mg/ml of BSA, 0.75 mM of MgCl_2_, 0.25 mM of each dNTPs, and milliQ water to complete a total reaction volume of 10 μL. For hair DNA samples with concentration lower than 10 ng/μL, or not measurable by spectrophotometer, we used 2 μL of the total volume of extracted DNA.

PCRs were carried out on a Thermal Cycler Eppendorf Mastercycler Gradient (Eppendorf AG, Hamburg, Germany) under the following conditions: an initial cycle of denaturation at 94°C for 5 min, followed by 30 cycles of 30 sec at 94°C, 45 sec at a locus-specific annealing temperature (Table 1) and 45 sec at 72°C; and by 8 cycles of 30 sec at 94ºC, 45 sec at 53ºC (M13 primer annealing temperature) and 45 sec at 72º C; with a final extension step at 72º for 10 min.

The methodology proposed by Schuelke [29] was employed for later identification of alleles from combined multiple loci using four different fluorophores (FAM, VIC, NED and PET) (Table 1). The amplified fragments were first visualized on an 1% agarose gel with 2 μL of each sample plus 1 μL of GelRed and 1 μL of bromophenol blue, under UV using a transilluminator (ETX-35.M, Vilmer Lourmat, Collégien France). Then, PCR products were genotyped on an automatic Sequencer 3730XL DNA Analyzer (Applied Biosystems, Waltham, Massachusetts, USA).

### Data analysis

The electropherograms were analysed with Geneious 6.0.6 [30] for identification of alleles and genotypes. Then, we checked stutters, allele dropout and the occurrence of null alleles using Micro-Checker 2.2.3 [31]. The Polymorphic Information Content (PIC) was estimated through the Cervus 3.0.3 program [32]. The number of alleles per locus (A_N_), number of effective alleles (A_E_) and expected (H_E_) and observed (H_O_) heterozygosity for each population were estimated with GenAlEx 6.51b2 [33]. Allelic richness (A_R_) and the inbreeding coefficients (F_IS_) were determined using the FSTAT 2.9.3 software [34]. P values for excess and deficit of heterozygotes and deviations from the Hardy-Weinberg Equilibrium (HWE) were tested with Genepop 4.0.10 [35].

P values for significant differences (P < 0.05) were determined based on the analysis of variance (ANOVA) and Students’s *t* test, after verifying sampling normality and homogeneity, using the Shapiro-Wilk and Levene tests, respectively, in R 4.2.1 [36]. To compare the genetic diversity parameters (H_O_, H_E_, A_N_) estimated for the European and the Brazilian captive populations of *L*. *chrysomelas*, we considered only the common homologous loci used in both studies.

Population differentiation was investigated through principal coordinate analysis (PCoA) and Bayesian clustering using GenAlEx 6.51b.2 [33] and Structure 2.1 [37], respectively. The number of most likely genetic groups (K) was determined by Structure Harvester 0.6.94 [38]. Five replicates were considered for each run, with K values ranging from 1 to 6, and using the admixture model with 200,000 MCMC interactions after a burn-in period of 40,000 iterations. The fixation index (F_ST_) [39] between populations was calculated using Arlequin 3.0 [40].

We also carried out a predictive analysis with Bottlesim 2.6 [41], to evaluate the effects on H_E_, H_O_ and A_N_ for simulated population size reductions of 0%, 20% and 50% in the next 100 years. The analysis was implemented with 1,000 iterations and considered complete overlapping of generations, dioecious reproduction, a 1:1 sex ratio, and constant population size. We also assumed that *L*. *chrysomelas* reaches sexual maturity at 2 years of age [42, 43] and that its lifespan is 16 years [44].

For the prediction analysis, we used the allele frequencies and the calculated effective population sizes (Ne). Ne values were estimated using the linkage disequilibrium through the NeEstimator 2.0 software [45]. We determined Ne for FPZSP and CPRJ populations, separately, and together (FPZSP-CPRJ). Ne is defined as being the size of an ideal population that undergoes the same amount of genetic drift as the population analysed, either by assessing the allele frequency or by the inbreeding rate [46]. Ne-based analyses are relevant, since the effective population size is a more accurate estimate for the population status than the census size, and, therefore, may reveal a more realist response on the heterozygosity and allele behaviour over generations [47].

## Results

Overall, we did not find differences in amplification rates between DNA from hair and blood samples (S1 Table). The percentage of unsuccessful amplification per locus (S1 Table) ranged from 1% (Lchu5) to 8.6% (Lchu4 and Leon27) for most loci, except for Lchu8 (27.9%). Stutters or allele dropout were not observed, while null alleles were indicated only for the locus Leon30. We found significant deviation from Hardy Weinberg Equilibrium in CPRJ for the loci Lchu5, Lchu9 and Leon30 (Table 2). However, genetic diversity (H_O_, H_E_, A_N_, A_R_, F_IS_; P < 0.05) and structure estimates were not altered when we removed these loci (S3 and S6 Tables, S3 Fig), therefore, they were not excluded from the analyses.

**Table 2:** Genetic diversity estimates for *Leontopithecus chrysomelas* captive groups from the Primatology Center of Rio de Janeiro (CPRJ, N = 55) and the Zoological Park Foundation of São Paulo (FPZSP, N = 49).

Locus	N	A_N_	A_R_	H_O_	H_E_	F_IS_	*P*_DH_	*P*_EH_	*P*_HWE_	PIC	A_P_
**Primatology Center of Rio de Janeiro**											
Lchu1	51	3	3.00	0.67	0.58	-0.157	0.893	0.109	0.155	0.51	-
Lchu3	52	6	5.96	0.83	0.76	-0.091	0.685	0.317	0.149	0.72	-
Lchu4	48	5	4.39	0.50	0.40	-0.247	1.000	0.017	0.515	0.36	-
Lchu5	48	6	5.60	0.77	0.76	-0.013	0.259	0.742	0.003*	0.72	290
Lchu9	53	5	4.91	0.70	0.73	0.037	0.015	0.986	0.000*	0.68	428
Lchu8	46	8	7.23	0.67	0.62	-0.093	0.561	0.445	0.430	0.59	226/232/240
Lchu6	54	5	4.90	0.61	0.66	0.077	0.146	0.857	0.092	0.62	182/186/192
Leon2	51	4	4.00	0.77	0.67	-0.143	0.603	0.029	0.081	0.60	-
Leon21	46	4	3.95	0.46	0.58	0.211	0.033	0.969	0.184	0.49	300/304
Leon27	50	3	3.00	0.68	0.55	-0.241	0.973	0.029	0.059	0.49	216
Leon30	55	7	6.48	0.55	0.72	0.237	0.007	0.993	0.000*	0.68	260/268
**Mean**			**4.86**	**0.65**	**0.64**	**-0.016**				**0.59**	
**Zoological Park Foundation of São Paulo**											
Lchu1	47	3	3.00	0.51	0.64	0.200	0.023	0.978	0.046	0.57	-
Lchu3	46	6	6.00	0.80	0.82	0.018	0.504	0.497	0.020	0.79	-
Lchu4	46	6	5.56	0.46	0.47	0.036	0.457	0.598	0.492	0.45	412
Lchu5	46	6	6.00	0.70	0.77	0.098	0.014	0.987	0.012	0.74	274
Lchu9	46	4	3.98	0.30	0.29	-0.048	0.749	0.545	0.537	0.28	-
Lchu8	29	5	5.00	0.72	0.69	-0.047	0.084	0.917	0.060	0.63	-
Lchu6	48	2	2.00	0.42	0.50	0.165	0.176	0.935	0.257	0.38	-
Leon2	45	4	3.96	0.60	0.59	-0.023	0.601	0.447	0.960	0.50	-
Leon21	41	2	2.00	0.44	0.34	-0.281	1.000	0.090	0.165	0.28	-
Leon27	45	3	3.00	0.64	0.61	-0.064	0.715	0.286	0.035	0.54	210
Leon30	44	5	4.51	0.80	0.44	-0.087	0.762	0.329	0.651	0.39	-
**Mean**			**4.09**	**0.55**	**0.56**	**0.025**				**0.50**	

N: sample number; A_N_: number of alleles; A_R_: allelic richness; H_O_: observed heterozygosity, H_E_: expected heterozygosity F_IS_: Inbreeding coefficient due to deviation from Hardy-Weinberg, *P*_DH_: p-values for the deficit of heterozygotes for the inbreeding coefficient F_IS;_ *P*_EH_: p-values for the excess of heterozygotes for the inbreeding coefficient F_IS_, *P*_HWE_: p-value for the Hardy Weinberg equilibrium; PIC: Polymorphic Information Content; A_P_: private alleles.

*Statistically significant values;

- Absence of private alleles.

The PIC values mostly ranged from 0.36 to 0.72 for CPRJ; and from 0.28 to 0.79 for FPZSP, with a mean of 0.59 and 0.50, respectively (Table 2), showing that the panel of microsatellites used herein is overall highly informative for both Brazilian captive populations of *L*. *chrysomelas*, according to the classification proposed by Botsein et al. [48], in which PIC>0.5 is highly informative, 0.25<PIC<0.50 reasonably informative, and PIC<0.25 is minimally informative.

The total number of alleles ranged from three to eight for CPRJ, and from two to six for FPZSP (Table 2). The values estimated for the mean allelic richness (A_R_), the number of private alleles (A_P_), observed heterozygosity (H_O_) and expected heterozygosity (H_E_) were higher in CPRJ when compared to FPZSP (Table 2). The CPRJ captive group showed a negative value of the mean inbreeding coefficient, indicating excess of heterozygotes; while the FPZSP showed a positive value, indicating deficit of heterozygotes (Table 2).

**Fig 1:**
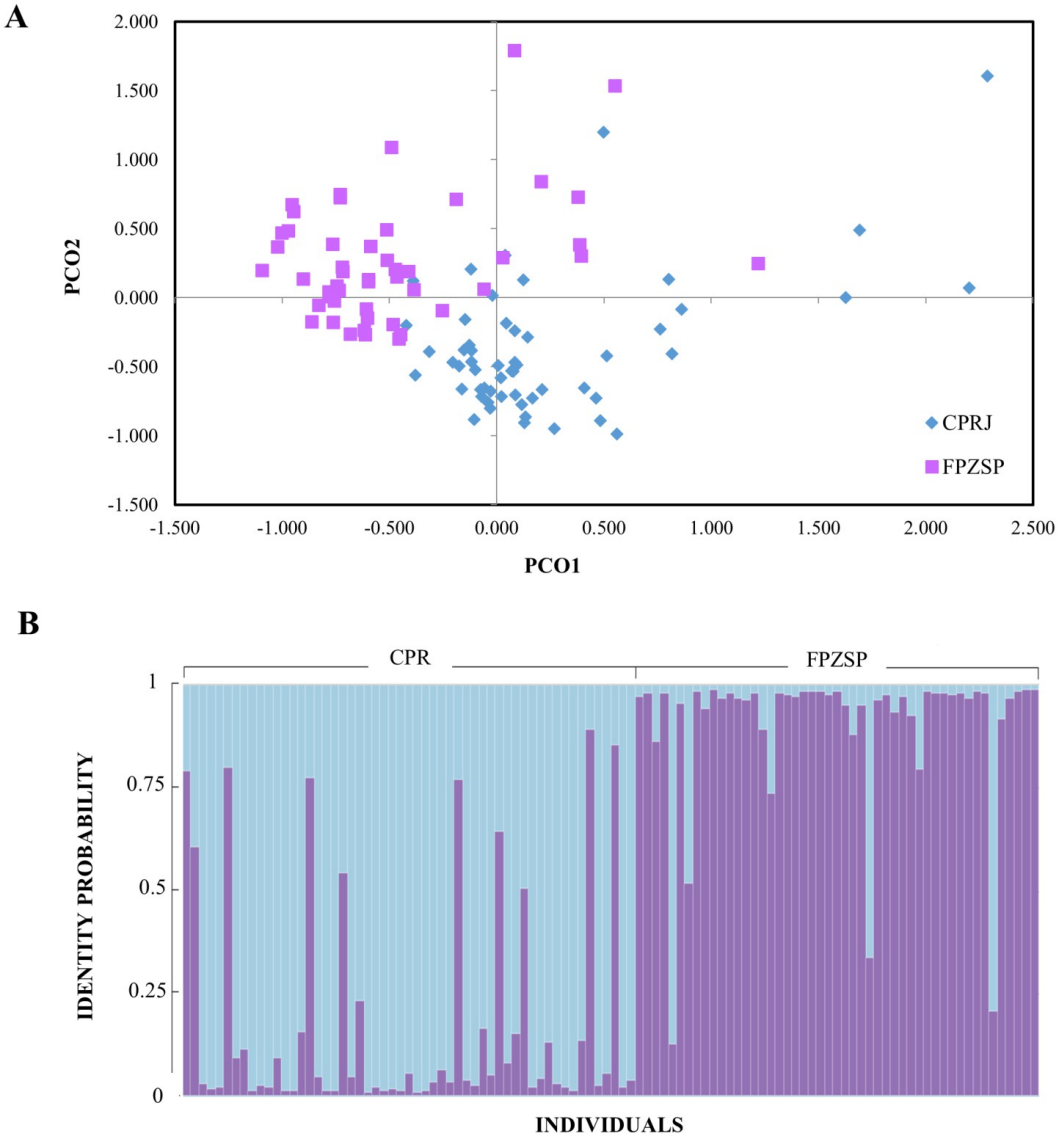
Population differentiation analyses for 104 individuals of *Leontopithecus chrysomelas* from the Primatology Center of Rio de Janeiro (CPRJ) and the Zoological Park Foundation of São Paulo (FPZSP). (A) Principal coordinate analysis (PCoA), showing the scores on the first (PCO1) and second (PCO2) principal coordinate. (B) Structure analysis results for CPRJ and FPZSP captive populations, considering the most probable K value (K = 2).

The principal coordinate (Fig 1A) and the Bayesian (Fig 1B) analyses distinguished both CPRJ and FPZSP populations, evidencing two main genetic groups (K = 2), confirmed by both Ln’(K) and Delta K estimates as shown in the (S2 Table and S2 Fig). The mean value of F_ST_ calculated for the CPRJ and FPZSP was 0.13182 (P = 0.000), indicating a moderate differentiation between populations.

**Fig 2:**
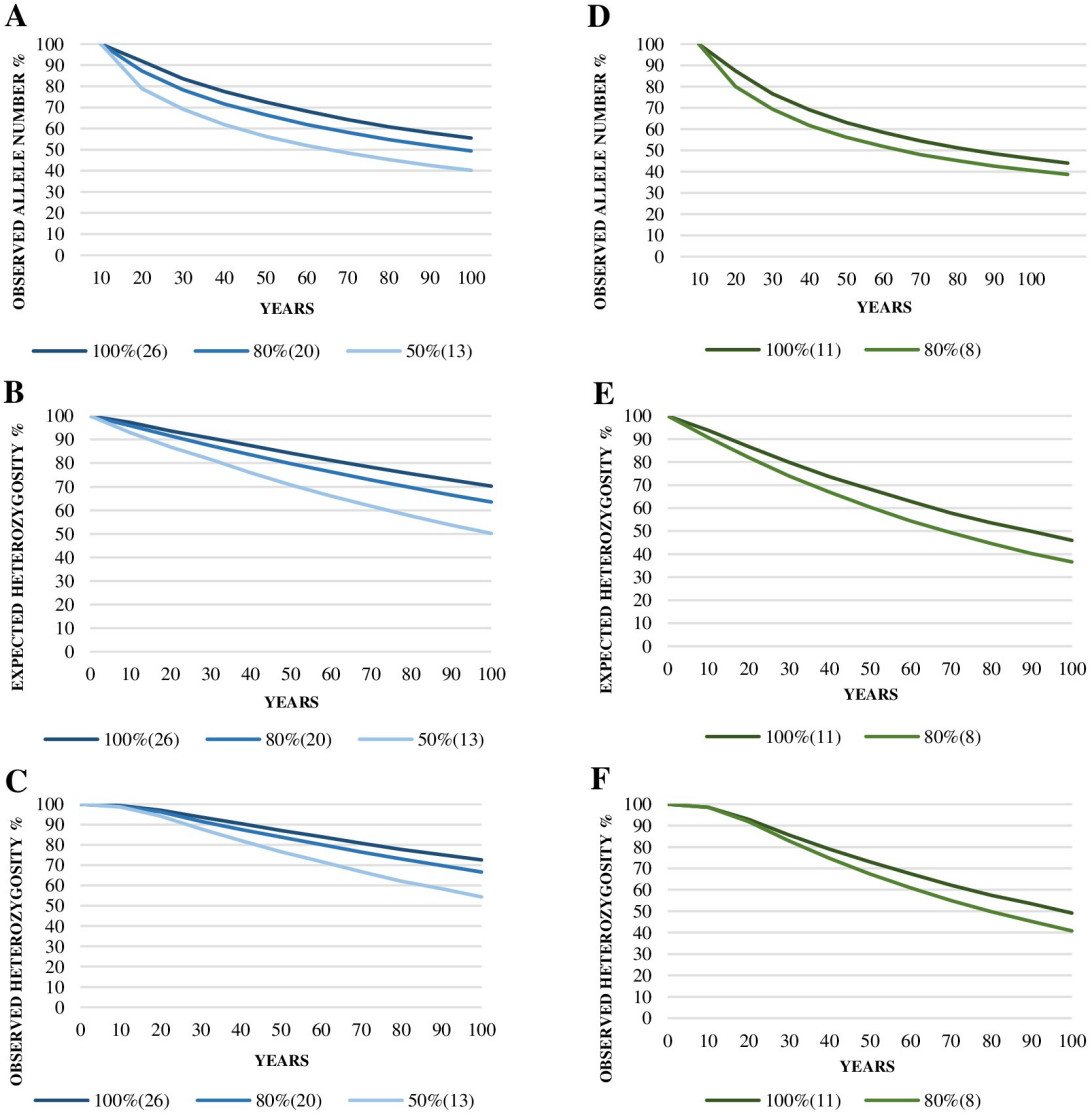
Prediction analysis for observed allele number, expected heterozygosity and observed heterozygosity reductions of the Brazilian *ex situ* populations of *Leontopithecus chrysomelas* from the Primatology Center of Rio de Janeiro (CPRJ) (A-C) and the Zoological Park Foundation of São Paulo (FPZSP) (D-F), for the next 100 years, using 100% of the effective population sizes (CPRJ: Ne = 26; FPZP: Ne = 11), and botlenecks of 20% and 50%.

The predictive analysis for population reduction events, considering the effective population sizes (Ne) for CPRJ (Ne = 26) and FPZSP (Ne = 11) (S4 and S5 Tables), showed that the expected and observed heterozygosity, as well as the number of alleles will drop below 90% in both captive populations over 100 years. Overall, the loss of genetic diversity in the number of alleles was greater than in the heterozygosity. The smaller the population, the more pronounced is the genetic diversity loss across generations. The decrease in allelic diversity, expected heterozygosity and observed heterozygosity was greater in FPZSP. When considering a 50% population size reduction, genetic diversity will rapidly decrease in CPRJ, and it will collapse in FPZSP. Considering no bottleneck events, the predictive analysis shows that both populations will retain more than 90% of allele number in the next 10 (FPZSP) and 20–30 (CPRJ) years. For the expected heterozygosity, more than 90% of it will be retained in the next 20–30 and 30–40 years in FPZSP and CPRJ, respectively (Fig 2, S5 Table).

**Fig 3:**
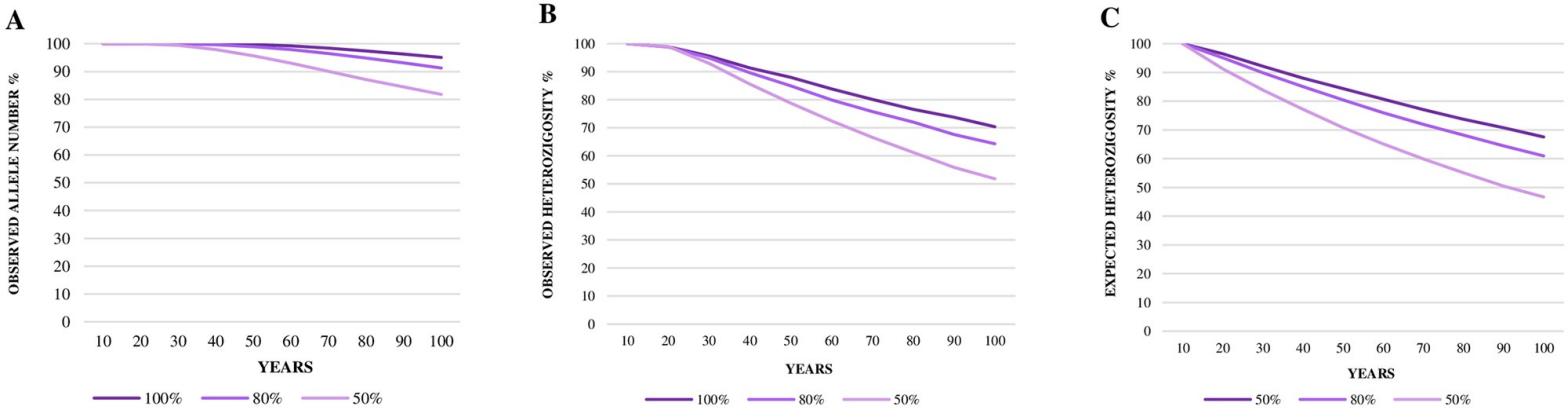
Prediction analysis for observed allele number, expected heterozygosity and observed heterozygosity reductions of the Brazilian *ex situ* population of *Leontopithecus chrysomelas* from the Primatology Center of Rio de Janeiro (CPRJ) and the Zoological Park Foundation of São Paulo (FPZSP), analyzed as a single population, for the next 100 years, using 100% of the effective population size (CPRJ-FPZP, Ne = 20), and botlenecks of 20% and 50%.

When we considered the two populations together (FPZSP-CPRJ), the effective population size was 20 (S4 Table); and the observed and expected heterozygosity showed tendency to decrease faster than the number of alleles (Fig 3, S5 Table). Considering bottleneck events, merely 40–60% of the current observed and expected heterozygosity will be maintained in the next 100 years. On the other hand, if the FPZSP-CPRJ population maintains its effective size, without bottleneck, more than 90% of the number of alleles and approximately 70% of the observed and expected heterozygosity will be retained over the years (Fig 3, S5 Table).

## Discussion

Overall, our findings suggest that the *ex situ* population of *L*. *chrysomelas* from the CPRJ shows a better capacity to maintain genetic diversity for a longer period under possible bottlenecks scenarios. In addition, such results highlight the importance of both FPZSP and CPRJ groups for the *ex situ* metapopulation management and point to the need for further *ex situ* and *in situ* integrated conservation programs.

The CPRJ and FPZSP populations evidenced genetic structuring among them, with moderate (F_ST_ = 0.132) differentiation [39], though some individuals from CPRJ showed a higher likelihood of belonging to the FPZSP cluster and vice versa. This later result is likely related to the management practices of these captive populations that include the exchange of individuals between institutions [49, 50]. Of note, CPRJ evidenced 13 private alleles, while FPZSP exhibited just three (see Table 2); from this total of 16, six and two are rare alleles (<5%), respectively (see S1 Table), suggesting that differences between populations may be specially related to the loss of rare alleles.

As rare alleles contribute relatively little to the population heterozygosity [51], their loss did not affect the heterozygosity levels much [51], as pointed below. In addition, FPZSP showed a positive but small value for the mean inbreeding coefficient (F_IS_ = 0.025), while CPRJ showed a mean negative inbreeding coefficient (F_IS_ = - 0.016), suggesting the management in the captive populations have been preventing the increase in homozygosity, which can lead to exposure of deleterious recessive alleles and compromise fitness traits such as those related to reproduction [6].

It is well known that the correlation between fitness and levels of genetic variation can be weak or even non-existent when assessed by neutral molecular markers, mainly for disregarding additive genetic diversity and the benefits caused by the purging of deleterious alleles (e.g., [47]). In this sense, homozygosity could be beneficial by eliminating harmful alleles from populations and, therefore, inbred populations with low heterozygosity would have adaptive advantages (e.g., [52, 53]). Notwithstanding, the Convention on Biological Diversity and the International Union for Conservation of Nature (IUCN) reaffirm the importance of considering genetic diversity to evaluate the threat status of species, based on the concept that small populations with lower heterozygosity levels and higher inbreeding rates are more prone to extinction [54]. In addition, meta-analyses compiling studies in different species have already shown that neutral genetic diversity can be correlated with fitness [47, 55, 56]. Also, a recent approach demonstrated that species with higher extinction risk status tend to have lower genetic diversity assessed by whole genome, mitochondrial and microsatellite analyses [57].

Further, despite the recent advances in genomics to assess non-neutral genetic diversity, most of the endangered and rare species have no assembled genome to perform robust analyses, mainly species from countries with high biodiversity and endemism, in addition to economic, social, and political concerns [58, 59]. Hence, the relevance of assessing heterozygosity and inbreeding levels in small and endangered species, by the use of neutral markers, to infer population viability is defended by the scientific community (e.g., [54, 56]). Thus, neutral genetic diversity has been widely assessed to infer on the general population health status [60], especially in the Neotropics (e.g., [18, 23, 24, 61–64]).

Our findings showed that genetic diversity estimates were similar between both CPRJ and FPZSP populations, and among these Brazilian populations and the captive population of *L*. *chrysomelas* from Europe (EUR) [19]. In addition, these estimates for *L*. *chrysomelas* were also similar to those reported for Brazilian and European captive populations of *L*. *chrysopygus* [23], as well as for wild populations of *Leontopithecus rosalia* [65], *Leontopithecus caissara* [66], *L*. *chrysopygus* [23] and *L*. *chrysomelas* [18].

Some of these earlier studies have used a very similar set of microsatellite markers and overall found low levels of heterozygosity (Table 3). Of note, EUR was analysed with nine homologous microsatellites, of which seven were also used in the present study. Considering only the common homologous loci employed in both studies, CPRJ and FPZSP exhibit a higher number of alleles than the EUR group (Table 4). Such findings may be due to the lower number of founders of the EUR population, and subsequent genetic drift effects [20]. However, the difference might be related to the difference in sampling numbers. Furthermore, the number of alleles and the values of heterozygosity are not significantly different (*P*_Na_ = 0.342, *P*_He_ = 0.83, *P*_Ho_ = 0.219) among the three captive groups.

**Table 3:** Comparison of mean genetic diversity values estimated for *Leontopithecus* spp through microsatellite analyses.

	Population	Microsatellite Loci	N	A_N_	A_R_	H_O_	H_E_	Reference
***L*. *chrysomelas* (Brazil-Captive)**	**CPRJ**	Lchu1, Lchu3, Lchu4, Lchu5, Lchu6, Lchu8, Lchu9, Leon2, Leon21, Leon27, Leon30	55	5.091	4.857	0.654	0.637	Present study
	**FPZSP**		49	4.182	4.091	0.552	0.560	
***L*. *chrysomelas* (Europe-Captive)**	**EUR**	Lchu1, Lchu2, Lchu3, Lchu4, Lchu5, Lchu6, Lchu7, Lchu8, Lchu9	29	3.670	-	0.630	0.590	[19]
***L*. *chrysomelas* (Wild)**	**Ilheús**	Lchu1, Lchu3, Lchu4, Lchu5, Lchu6, Lchu8, Lchu9, Leon2, Leon21, Leon27, Leon30	17	3.5	1.5	0.5	0.5	[18]
	**Teimoso**		7	2.3	1.4	0.4	0.4	
	**Araraúna**		84	5.3	1.6	0.5	0.6	
	**Barro Branco**		6	3.5	1.7	0.6	0.7	
***L*. *chrysopygus* (Captive)**	**FPZSP**	Lchu1, Lchu6, Lchu7, Lchu8, Leon2, Leon15, Leon21, Leon3c75, Leon31c97, Leon30c73, Leon35c42, Leon11c72, LrP2BH6, Lr.P5BE6, Lr.P3AF1	20	2.267	2.143	0.697	0.462	[23]
	**CPRJ**		17	2.333	2.205	0.757	0.461	
	**DWCT**		16	2.000	1.951	0.715	0.410	
***L*. *chrysopygus* (Wild)**	**Capão Bonito**		10	2.000	1.974	0.673	0.403	[23]
***L*. *rosalia* (Wild)**	**Poço das Antas**	LrP2BH6, LrP2BA2, LrP5BG3, LrP5BE6, LrP3AF1	27	3.8	-	0.65	0.66	[65]
	**SJ**		16	3.0	-	0.55	0.56	
	**LB**		8	2.3	-	0.34	0.53	
	**Bauen**		6	2.0	-	0.43	0.42	
***L*. *caissara* (Wild)**	**Ariri**	Leon2, Leon3c20, Leon15c85, Leon21c75, Leon30c73, Leon31c97, LrP2BH6, Lchu4, Lchu7	52	2.56	2.325	0.55	0.42	[66, 67]
	**Superagui Island**		34	2.67	2.534	0.56	0.48	

N: number of individuals analysed, A_N_: allele number, A_R_: allelic richness, H_O_: Observed heterozygosity, H_E_: expected heterozygosity. The Primatology Center of Rio de Janeiro (CPRJ) and the Zoological Park Foundation of Sao Paulo (FPZSP).

**Table 4:** Comparison of mean genetic diversity parameters estimated for the *ex situ* populations of *Leontopithecus chrysomelas* from the Primatology Center of Rio de Janeiro (CPRJ), the Zoological Park Foundation of Sao Paulo (FPZSP), and Europe (EUR), considering only seven homologous microsatellite loci.

Population	Loci	N	A_N_	H_O_	H_E_
**CPRJ**	*Lchu1*, *Lchu3*, *Lchu4*, *Lchu5*, *Lchu6*, *Lchu8*, *Lchu9*	55	5.429	0.678	0.643
**FPZSP**	*Lchu1*, *Lchu3*, *Lchu4*, *Lchu5*, *Lchu6*, *Lchu8*, *Lchu9*	49	4.571	0.559	0.598
**EUR**	*Lchu1*, *Lchu3*, *Lchu4*, *Lchu5*, *Lchu6*, *Lchu8*, *Lchu9*	29	4	0.62	0.61

N: number of individuals analysed, A_N_: number of alleles, H_O_: observed heterozygosity and H_E_: expected heterozygosity.

Regarding the Brazilian captive populations of *L*. *chrysomelas* studied herein and the *L*. *chrysopygus* ones molecularly studied by Ayala-Burbano *et al*. [23], both species presented values of observed heterozygosity in general higher than expected heterozygosity, and despite the positive mean inbreeding coefficient observed in FPZSP for *L*. *chrysomelas*, the majority of the used microsatellite loci showed negative inbreeding coefficients (see Table 2). However, Ayala-Burbano *et al*. [23, 24] mention that, although the captive populations of *L*. *chrysopygus* do not present heterozygosity deficiency, the individuals are highly related, as probably is the case of the captive populations of *L*. *chrysomelas* here analysed. Negative F_IS_ values are quite common in captivity and have been also reported for other species, such as the red panda [68], the Chinese water deer [69], the Arabian sand cat [70] and the bearded vulture [71].

Comparing both Brazilian captive populations with wild populations of *L*. *chrysomelas* studied by Moraes *et al*. [18], the observed and expected heterozygosity showed similar values (Table 3). However, the number of alleles is higher in both CPRJ and FPZSP than that observed in wild populations, except for the Araraúna population. Since differences in the number of alleles are sensitive to the number of individuals analysed [18, 72], this result may be linked to the differences in sample sizes [56].

Indeed, allelic richness has been considered an efficient estimator to measure genetic diversity [17, 55] and evaluate the long-term evolutionary potential of a population and consequently its conservation status [73–76]. Within a study, in order to take into account, the difference in sample size, allelic richness values are based on the smallest sample size [77]. However, as this size varies among studies (from 6 to 49; see Table 3) it remains hard to compare allelic richness values across studies. It probably explains why the sampled wild population from Araraúna, with a high number of analysed individuals (N = 84), showed lower allelic richness than the captive populations studied herein.

The wild *L*. *chrysomelas* populations in general have larger population sizes [44]. However, according to a prior population viability analysis [78], Araraúna is the only population of the species in nature that is currently found in a protected forest area (RPPN, Private Reserve of Natural Heritage) and has the capacity to retain a high density and adequate levels of remaining genetic diversity over time. Further, most of the wild populations of *L*. *chrysomelas* presented higher proportions of private alleles [18] than that observed in CPRJ and FPZSP (see Table 2). Such results may be related to the high degree of fragmentation in nature which often difficult dispersion and gene flow among unconnected populations [18], accentuating the genetic drift effects and consequent population differentiation [73]. On the other hand, in captivity, the management has been including exchanges of individuals between institutions, aiming to form less or un-related mate-pairs, and thus retain genetic diversity and avoid an increase in inbreeding rates [50, 73]. Despite this, the recommendations for establishing mate-pairs do not always result effectively in feasible reproduction in *ex situ* conditions.

Captive breeding programs generally include family groups formed by selected mate-pairs and their descendants [6]. Nevertheless, even when multiple couples are selected as potential breeders, not all of them copulate and/or generate viable offspring. Thereby, captive offspring often come from a few couples that in general repeatedly contribute to population growth over time [6, 73], explaining the values of Ne found herein (see S4 Table). Thus, the genetic structure and diversity of captive populations usually is strongly related to the origin of the parents, their kinships, and the inbreeding rates over generations. In addition, random effects of genetic drift are generally accentuated in small and closed populations such as many *ex situ* populations [73]. Also, although the introduction of individuals from nature may occur, these wild animals can be related to each other and/or never become breeders, as such not increasing the genetic diversity over generations [15].

According to Ballou *et al*. [15] and Ballou & Mace [21], the whole *ex situ* metapopulation of GHLT was initially formed by 83 founders (69 in South America), and also a contribution of 96 founders from the illegal exports of 1984 and 1985, therefore, a high number compared to the *ex situ* population of *L*. *chrysopygus* [23, 24]. Despite this, our prediction analysis, based on the effective population sizes, indicates that both CPRJ and FPZSP will not be able to retain adequate levels of genetic diversity (90%) after the next 40 and 10 years, respectively (see Fig 2). Even without bottlenecks, genetic diversity will drastically decrease in both populations, especially in FPZSP (see S5 Table). Further, when we considered both CPRJ and FPZSP *ex situ* groups of *L*. *chrysomelas* as a single population, they together will not be able to retain 90% of the current observed and expected heterozygosity in the next 40–50 years, even without bottleneck events (see Fig 3, S5 Table). On the other hand, the number of alleles will be maintained at a high level (80–90%). Such result may be related to the fact that when both populations are considered as a single one, the observed heterozygosity and the effective population size decrease, despite the allele richness and the frequency of private alleles being higher than in each Brazilian captive population (see S5 Table).

Indeed, CPRJ and FPZSP present different private alleles, and the proportion of these alleles and the allele richness are much higher in CPRJ than in FPZSP, which evidenced higher homozygosity and faster loss of genetic diversity than CPRJ. Therefore, the presence of some private alleles might contribute to maintaining the allele number over time in FPZSP-CPRJ, since the specific alleles from each population contribute to the total allele diversity [79, 80]. Furthermore, the levels of heterozygosity will decrease faster probably due to the increase in inbreeding because of the small population size. Thus, although populations with higher levels of allele richness in general show a tendency to lose alleles more quickly, the loss in heterozygosity may be faster than in number of alleles in inbred populations, as those from endangered species [81].

### Final considerations

The simulated scenario of population viability reductions is already a reality for the *ex situ* groups of *L*. *chrysomelas*, since the captive individuals are aging and few animals have regularly been reproducing [22]. In 2011, for example, FPZSP kept more than 70 GHLTs under human care, however, its population size has been reduced by about 70%, in order to avoid surplus animals due to a lack of available enclosures, and currently there is only one adult female able for reproduction, as the others are already elderly (Monticelle C., personal communication). In the CPRJ, nowadays, there is only one mate-pair regularly breeding, which recently produced five cubs as follows: one in 2018, three in 2021 and one in 2022; and the captive individuals are old and/or closely related as well (Pissinatti A., personal communication).

For the congeneric *L*. *chrysopygus*, a previous similar genetic study performed in *ex situ* populations detected a moderate genetic differentiation between the Brazilian groups and between the Brazilian and European captive populations [23]. However, after posterior management actions, no genetic structuring was detected, even considering all related animals and family groups of the whole *ex situ* metapopulation of the species (Freitas P.D., personal communication). Furthermore, the introduction of confiscated wild *L*. *chrysopygus* in captivity allowed the formation of new mate-pairs and the generation of viable offspring after a long period of population decline [24, 82]. More recently, translocations of animals from both Brazilian captive populations to Europe also resulted in reproduction and viable offspring after about eight years without any birth at the Jersey Zoo [82].

Therefore, for the *ex situ* population of *L*. *chrysomelas* we suggest new exchanges of captive individuals, especially from the particular family groups of both CPRJ and FPZSP institutions, and EUR as well; and, if possible, an extra effort for the formation of mate-pairs between these and new confiscated wild individuals introduced in captivity. It is noteworthy that currently there are smaller GHLT groups under human care in 15 other institutions in Brazil (Galbusera P., personal communication) which continue to be included in the *ex situ* management. In addition, semen analysis and cryopreservation, as tested in the FPZSP [83] an alternative tool for contributing to the maintenance of genetic diversity in the future [6, 84].

Furthermore, in addition to the pedigree analyses, in order to estimate the genetic diversity and inbreeding [22], mainly using the PMx software [85], we also suggest that standardized molecular-genetic analyses of the whole *ex situ* population of *L*. *chrysomelas* (including the Brazilian and European groups) be carried out from now on, aiming for a better comparison of genetic parameters (e.g. allelic richness, see above) and their optimized use for management purposes [24]. Likewise, it will be important to evaluate both *ex situ* and *in situ* populations in a standardized way, to allow an integrated management of the species according to the One Plan Approach proposed by the IUCN Species Survival Commission [86]. Therefore, in addition to using the same microsatellite loci panel, allele and genotype scorings must be performed in a single-integrated approach. Further, genomic tools, based on next-generation sequencing and large-scale data analysis, such as Genotyping by Sequencing, GBS, [87] and double digest Restriction-site Associated DNA, ddRAD, [88], must be applied with the goal of detecting non-neutral loci and then searching for genomic signatures of differential selection and adaptive genetic diversity [89].

## Supporting information

S1 Table Information related to identification number (sample), institutions where captive animals were kept, sex and type of tissue collected, for the 104 samples from both captive populations of *Leontopithecus chrysomelas* studied using a panel of 11 microsatellite loci (Lchu1, Lchu3, Lchu4, Lchu5, Lchu6, Lchu8, Lchu9, Leon2, Leon21, Leon27, Leon30) and their percentage of unsuccessful amplification (NA). CPRJ: Primatology Center of Rio de Janeiro; FPZSP: Zoological Park Foundation of São Paulo. *Rare alleles (<5%).
(DOCX)

S2 Table Values of Ln’(K) and Delta K for the Structure analysis measured with Structure Harvester for the Brazilian captive *Leontopithecus chrysomelas* populations using a panel of 11 microsatellite loci (Lchu1, Lchu3, Lchu4, Lchu5, Lchu6, Lchu8, Lchu9, Leon2, Leon21, Leon27, Leon30). K: number of genetic clusters.
(DOCX)

S3 Table Values of Ln’(K) and Delta K for the Structure analysis measured with Structure Harvester for the Brazilian captive *Leontopithecus chrysomelas* populations using only the eight microsatellite loci with no HWE deviations (Lchu1, Lchu3, Lchu4, Lchu6, Lchu8, Leon2, Leon21, Leon27). K: number of genetic clusters. HWE: Hardy Weinberg Equilibrium.
(DOCX)

S4 Table Summary of the effective population size (Ne) and confidence interval (95% CI) values, estimated with NeEstimator 2.0 software, for the Brazilian captive populations of *Leontopithecus chrysomelas*. CPRJ: Primatology Center of Rio de Janeiro; FPZSP: Zoological Park Foundation of São Paulo.
(DOCX)

S5 Table Comparison of mean genetic diversity values for the Brazilian captive *Leontopithecus chrysomelas* populations retained over 100 years for the simulations of 0%, 20% and 50% reductions in size of the populations of the Zoological Park Foundation of São Paulo (FPZSP), the Primatology Center of Rio de Janeiro (CPRJ) and the FPZSP-CPRJ. Ne: effective population sizes, A_N_: number of alleles, H_O_: observed heterozygosity and H_E_: expected heterozygosity.
(DOCX)

S6 Table Summary of the P values for differences between the genetic diversity parameters (GD) of the Brazilian captive populations of *Leontopithecus chrysomelas*, using the total panel of 11 microsatellite loci (Lchu1, Lchu3, Lchu4, Lchu5, Lchu6, Lchu8, Lchu9, Leon2, Leon21, Leon27 and Leon30) and the reduced panel of eight microsatellite loci (Lchu1, Lchu3, Lchu4, Lchu6, Lchu8, Leon2, Leon21 and Leon27), after exclusion of loci with a significant HWE deviation. CPRJ: Primatology Center of Rio de Janeiro; FPZSP: Zoological Park Foundation of São Paulo. A_R_: alleles richness, H_E_: expected heterozygosity, H_O_: observed heterozygosity, F_IS_: inbreeding coefficient. HWE: Hardy Weinberg Equilibrium. * Significant statistically differences P < 0.05.
(DOCX)

S1 Fig Illustrative map showing (in red) the locations in Brazil where the *ex situ* populations of *Leontopithecus chrysomelas* are under human care and the respective institution names; and (in blue) the locations of the *in situ* populations that have already been studied by microsatellite data. CPRJ: Primatology Center of Rio de Janeiro; FPZSP: Zoological Park Foundation of São Paulo.
(DOCX)

S2 Fig Graph showing the values of DeltaK = mean(|L’’(K)|) / sd(L(K)) obtained by Structure and Structure Harvester for the Brazilian captive populations of *Leontopithecus chrysomelas*, indicating the number of genetic clusters (K). CPRJ: Primatology Center of Rio de Janeiro; FPZSP: Zoological Park Foundation of São Paulo.
(DOCX)

S3 Fig Structure analysis results for 104 individuals of *Leontopithecus chrysomelas* from the Primatology Center of Rio de Janeiro (CPRJ) and the Zoological Park Foundation of São Paulo (FPZSP), using only the eight microsatellite loci in HWE (Lchu1, Lchu3, Lchu4, Lchu6, Lchu8, Leon2, Leon21and Leon27), considering the most probable K value (K = 2). HWE: Hardy Weinberg Equilibrium.
(DOCX)
