# Supplementary material for: Genetic diversity in ex situ populations of the endangered Leontopithecus chrysomelas and implications for its conservation
Source: PLoS One. 2023 Aug 2;18(8):e0288097. doi: 10.1371/journal.pone.0288097 (PMC10395972; doi:10.1371/journal.pone.0288097)
Supplement: S1 Table — CPRJ: Primatology Center of Rio de Janeiro; FPZSP: Zoological Park Foundation of São Paulo. *Rare alleles (<5%). (DOCX) [file pone.0288097.s001.docx]

**S1 Table**. Information related to identification number (sample), institutions where captive animals were kept, sex and type of tissue collected, for the 104 samples from both captive populations of *Leontopithecus chrysomelas* studied using a panel of 11 microsatellite loci (Lchu1, Lchu3, Lchu4, Lchu5, Lchu6, Lchu8, Lchu9, Leon2, Leon21, Leon27, Leon30) and their percentage of unsuccessful amplification (NA). CPRJ: Primatology Center of Rio de Janeiro; FPZSP: Zoological Park Foundation of São Paulo. *Rare alleles (<5%).

| **Institution** | **Sample** | **Sex** | **Tissue** | **Lchu1** | | **Lchu3** | | **Lchu4** | | **Lchu5** | | **Lchu6** | | **Lchu8** | | **Lchu9** | | **Leon2** | | **Leon21** | | **Leon27** | | **Leon30** | |
| --- | --- | --- | --- | --- | --- | --- | --- | --- | --- | --- | --- | --- | --- | --- | --- | --- | --- | --- | --- | --- | --- | --- | --- | --- | --- |
| **CPRJ** | 1859 | M | blood | 210 | 214 | 316 | 332 | 396* | 400 | 278 | 282 | 180 | 190 | 226* | 236 | 420 | 426 | 218 | 220 | 302 | 302 | 212 | 214 | 270 | 270 |
| **CPRJ** | 2186 | F | blood | 210 | 214 | 316 | 316 | 400 | 408* | 278 | 282 | 182 | 190 | 226* | 236 | 420 | 426 | 218 | 220 | 302 | 302 | 214 | 214 | 270 | 270 |
| **CPRJ** | 2548 | F | blood | 214 | 214 | 332 | 340 | 400 | 400 | 262 | 262 | 180 | 190 | 226* | 230 | 420 | 426 | 218 | 218 | 302 | 302 | 212 | 214 | 270 | 270 |
| **CPRJ** | 2570 | M | blood | 214 | 214 | 332 | 332 | 400 | 404 | 262 | 286 | 182 | 182 | 226* | 230 | 420 | 426 | 218 | 220 | 302 | 302 | 212 | 214 | 270 | 270 |
| **CPRJ** | 2605 | M | blood | 214 | 214 | 336 | 340 | 400 | 400 | 262 | 262 | 180 | 182 | 0 | 0 | 0 | 0 | 218 | 218 | 302 | 302 | 214 | 214 | 270 | 270 |
| **CPRJ** | 2606 | M | blood | 210 | 214 | 316 | 332 | 400 | 400 | 278 | 282 | 180 | 190 | 230 | 236 | 420 | 426 | 218 | 220 | 302 | 302 | 214 | 214 | 270 | 270 |
| **CPRJ** | 2896 | M | blood | 214 | 218 | 332 | 336 | 400 | 404 | 262 | 286 | 180 | 190 | 230 | 236 | 420 | 426 | 0 | 0 | 302 | 302 | 214 | 214 | 270 | 270 |
| **CPRJ** | 1918 | M | blood | 210 | 214 | 332 | 336 | 396* | 400 | 286 | 298 | 190 | 190 | 230 | 234 | 420 | 426 | 218 | 224 | 298 | 302 | 214 | 214 | 260* | 272 |
| **CPRJ** | 2226 | M | blood | 214 | 214 | 332 | 340 | 400 | 404 | 286 | 286 | 182 | 190 | 230 | 230 | 420 | 428 | 220 | 224 | 0 | 0 | 214 | 216 | 258 | 272 |
| **CPRJ** | 2012 | M | blood | 214 | 214 | 336 | 340 | 400 | 404 | 262 | 298 | 190 | 190 | 230 | 230 | 420 | 426 | 220 | 220 | 298 | 300 | 214 | 216 | 266 | 270 |
| **CPRJ** | 2152 | F | blood | 214 | 218 | 336 | 340 | 400 | 404 | 262 | 262 | 190 | 190 | 230 | 230 | 420 | 426 | 220 | 222 | 298 | 300 | 214 | 216 | 266 | 270 |
| **CPRJ** | 2153 | F | blood | 210 | 214 | 328 | 332 | 400 | 404 | 278 | 282 | 190 | 190 | 230 | 230 | 420 | 420 | 220 | 222 | 300 | 300 | 214 | 214 | 258 | 270 |
| **CPRJ** | 2423 | M | blood | 214 | 214 | 332 | 336 | 400 | 400 | 262 | 286 | 186 | 190 | 230 | 230 | 420 | 424 | 222 | 224 | 0 | 0 | 0 | 0 | 268 | 272 |
| **CPRJ** | 2533 | F | blood | 214 | 218 | 336 | 340 | 400 | 400 | 262 | 286 | 182 | 186 | 230 | 230 | 420 | 428 | 224 | 224 | 298 | 298 | 214 | 216 | 268 | 268 |
| **CPRJ** | 2565 | M | blood | 214 | 214 | 332 | 332 | 400 | 404 | 282 | 298 | 180 | 190 | 230 | 234 | 424 | 424 | 220 | 224 | 298 | 298 | 214 | 214 | 270 | 270 |
| **CPRJ** | 2566 | M | blood | 214 | 218 | 324* | 328 | 400 | 400 | 0 | 0 | 180 | 190 | 230 | 234 | 424 | 424 | 220 | 224 | 298 | 298 | 214 | 214 | 270 | 270 |
| **CPRJ** | 2900 | F | blood | 210 | 210 | 328 | 336 | 400 | 400 | 0 | 0 | 182 | 186 | 230 | 236 | 420 | 428 | 224 | 224 | 298 | 298 | 214 | 216 | 268 | 268 |
| **CPRJ** | 2233 | M | blood | 0 | 0 | 0 | 0 | 0 | 0 | 0 | 0 | 182 | 192* | 230 | 240 | 424 | 428 | 220 | 224 | 302 | 304* | 212 | 214 | 270 | 272 |
| **CPRJ** | 2364 | F | blood | 214 | 218 | 332 | 332 | 400 | 400 | 282 | 298 | 180 | 192* | 230 | 240 | 424 | 424 | 220 | 224 | 300 | 302 | 214 | 216 | 268 | 272 |
| **CPRJ** | 2228 | M | blood | 214 | 218 | 336 | 340 | 400 | 400 | 282 | 298 | 180 | 190 | 230 | 230 | 420 | 426 | 220 | 220 | 298 | 298 | 214 | 214 | 266 | 270 |
| **CPRJ** | 2041 | F | blood | 214 | 214 | 332 | 340 | 400 | 400 | 262 | 282 | 190 | 190 | 230 | 234 | 424 | 424 | 220 | 224 | 0 | 0 | 212 | 214 | 270 | 270 |
| **CPRJ** | 2622 | F | blood | 210 | 214 | 316 | 332 | 400 | 404 | 282 | 286 | 190 | 190 | 230 | 234 | 424 | 424 | 220 | 224 | 298 | 302 | 214 | 214 | 270 | 270 |
| **CPRJ** | 2172 | M | blood | 214 | 218 | 332 | 336 | 400 | 400 | 262 | 286 | 182 | 190 | 240 | 240 | 424 | 428 | 220 | 220 | 298 | 304* | 212 | 216 | 272 | 272 |
| **CPRJ** | 2170 | F | blood | 210 | 214 | 336 | 340 | 400 | 416* | 262 | 262 | 180 | 182 | 236 | 238* | 424 | 424 | 220 | 224 | 298 | 304* | 212 | 214 | 270 | 272 |
| **CPRJ** | 2445 | M | blood | 210 | 214 | 340 | 340 | 400 | 400 | 262 | 286 | 182 | 190 | 230 | 240 | 424 | 424 | 220 | 224 | 298 | 302 | 214 | 216 | 258 | 270 |
| **CPRJ** | 2538 | M | blood | 210 | 214 | 328 | 340 | 400 | 400 | 262 | 286 | 182 | 182 | 230 | 238* | 422* | 428 | 224 | 224 | 298 | 302 | 212 | 214 | 258 | 272 |
| **CPRJ** | 2397 | M | blood | 214 | 218 | 336 | 340 | 400 | 400 | 262 | 262 | 182 | 182 | 230 | 238* | 422* | 422* | 220 | 224 | 302 | 302 | 212 | 214 | 258 | 272 |
| **CPRJ** | 2136 | M | blood | 210 | 214 | 328 | 340 | 400 | 404 | 282 | 286 | 0 | 0 | 0 | 0 | 0 | 0 | 0 | 0 | 298 | 302 | 214 | 216 | 258 | 270 |
| **CPRJ** | 2147 | F | blood | 214 | 214 | 332 | 340 | 400 | 400 | 262 | 286 | 182 | 190 | 0 | 0 | 420 | 428 | 222 | 224 | 298 | 302 | 214 | 216 | 268 | 272 |
| **CPRJ** | 2452 | M | blood | 214 | 214 | 332 | 336 | 400 | 400 | 298 | 298 | 182 | 186 | 0 | 0 | 420 | 428 | 222 | 224 | 298 | 302 | 214 | 214 | 272 | 272 |
| **CPRJ** | 2453 | F | blood | 210 | 214 | 316 | 340 | 400 | 400 | 282 | 286 | 182 | 190 | 0 | 0 | 420 | 428 | 222 | 224 | 298 | 302 | 214 | 214 | 272 | 272 |
| **CPRJ** | 1503 | M | blood | 214 | 214 | 332 | 332 | 400 | 404 | 286 | 286 | 190 | 190 | 0 | 0 | 420 | 424 | 220 | 224 | 302 | 302 | 212 | 212 | 270 | 270 |
| **CPRJ** | 1433 | F | blood | 214 | 218 | 332 | 340 | 400 | 404 | 262 | 286 | 190 | 190 | 0 | 0 | 420 | 424 | 224 | 224 | 298 | 302 | 212 | 214 | 270 | 274* |
| **CPRJ** | 2272 | M | blood | 210 | 218 | 324* | 332 | 400 | 400 | 0 | 0 | 190 | 190 | 0 | 0 | 420 | 420 | 0 | 0 | 298 | 302 | 212 | 212 | 270 | 274* |
| **CPRJ** | 2196 | M | blood | 214 | 218 | 336 | 340 | 400 | 400 | 262 | 262 | 180 | 190 | 0 | 0 | 426 | 426 | 220 | 222 | 298 | 298 | 212 | 214 | 270 | 274* |
| **CPRJ** | 2354 | M | blood | 214 | 218 | 336 | 340 | 400 | 404 | 262 | 286 | 182 | 190 | 232 | 236 | 420 | 428 | 222 | 224 | 298 | 302 | 214 | 216 | 266 | 270 |
| **CPRJ** | 2357 | F | blood | 214 | 218 | 328 | 332 | 400 | 400 | 278 | 286 | 190 | 190 | 230 | 234 | 424 | 424 | 220 | 224 | 298 | 298 | 214 | 214 | 258 | 272 |
| **CPRJ** | 2227 | F | blood | 210 | 218 | 332 | 336 | 400 | 400 | 282 | 286 | 182 | 190 | 230 | 230 | 420 | 428 | 220 | 224 | 298 | 302 | 214 | 216 | 266 | 270 |
| **CPRJ** | 2308 | F | blood | 214 | 218 | 324* | 332 | 400 | 400 | 278 | 278 | 190 | 190 | 230 | 234 | 420 | 424 | 220 | 224 | 298 | 302 | 212 | 214 | 270 | 272 |
| **CPRJ** | 2547 | F | blood | 214 | 214 | 332 | 332 | 396* | 400 | 282 | 298 | 190 | 190 | 230 | 230 | 420 | 426 | 224 | 224 | 302 | 302 | 212 | 214 | 270 | 274* |
| **CPRJ** | 2117 | F | blood | 210 | 210 | 328 | 340 | 400 | 404 | 282 | 286 | 186 | 190 | 230 | 236 | 420 | 424 | 220 | 224 | 0 | 0 | 214 | 216 | 270 | 270 |
| **CPRJ** | 2185 | M | blood | 210 | 214 | 336 | 340 | 400 | 400 | 282 | 286 | 180 | 190 | 230 | 236 | 420 | 426 | 220 | 220 | 298 | 298 | 0 | 0 | 258 | 270 |
| **CPRJ** | 2003 | M | blood | 214 | 214 | 332 | 336 | 400 | 400 | 262 | 286 | 186 | 186 | 230 | 236 | 420 | 424 | 222 | 224 | 298 | 302 | 214 | 216 | 266 | 270 |
| **CPRJ** | 1981 | F | blood | 214 | 218 | 332 | 340 | 400 | 404 | 262 | 286 | 180 | 190 | 230 | 236 | 420 | 426 | 220 | 222 | 298 | 298 | 212 | 214 | 258 | 272 |
| **CPRJ** | 2122 | M | blood | 214 | 214 | 332 | 336 | 400 | 404 | 262 | 282 | 180 | 180 | 230 | 230 | 420 | 426 | 220 | 222 | 298 | 298 | 212 | 214 | 266 | 266 |
| **CPRJ** | 1894 | M | blood | 210 | 214 | 336 | 336 | 0 | 0 | 262 | 286 | 190 | 190 | 230 | 230 | 426 | 426 | 220 | 224 | 302 | 302 | 212 | 214 | 270 | 270 |
| **CPRJ** | 2498 | M | blood | 214 | 218 | 332 | 336 | 400 | 404 | 262 | 298 | 182 | 182 | 236 | 238* | 420 | 424 | 220 | 224 | 298 | 302 | 212 | 214 | 258 | 270 |
| **CPRJ** | 2845 | M | blood | 214 | 218 | 332 | 336 | 400 | 416* | 282 | 290* | 186 | 190 | 230 | 230 | 420 | 428 | 220 | 224 | 0 | 0 | 0 | 0 | 272 | 272 |
| **CPRJ** | 2359 | F | blood | 214 | 218 | 332 | 332 | 0 | 0 | 282 | 282 | 190 | 190 | 230 | 230 | 420 | 424 | 220 | 224 | 0 | 0 | 212 | 214 | 270 | 270 |
| **CPRJ** | 2607 | F | blood | 210 | 218 | 328 | 332 | 400 | 404 | 278 | 282 | 180 | 190 | 230 | 234 | 420 | 424 | 220 | 224 | 298 | 298 | 212 | 214 | 270 | 270 |
| **CPRJ** | 2355 | F | blood | 210 | 214 | 332 | 340 | 400 | 404 | 0 | 0 | 186 | 190 | 230 | 236 | 420 | 424 | 220 | 224 | 298 | 298 | 214 | 216 | 268 | 272 |
| **CPRJ** | 1893 | M | blood | 0 | 0 | 316 | 324* | 0 | 0 | 282 | 286 | 180 | 192* | 230 | 240 | 424 | 424 | 224 | 224 | 0 | 0 | 214 | 214 | 268 | 268 |
| **CPRJ** | 1982 | M | blood | 210 | 210 | 328 | 340 | 0 | 0 | 282 | 286 | 180 | 190 | 230 | 236 | 420 | 426 | 220 | 222 | 0 | 0 | 0 | 0 | 266 | 270 |
| **CPRJ** | 2173 | F | blood | 0 | 0 | 0 | 0 | 0 | 0 | 0 | 0 | 190 | 190 | 228* | 230 | 426 | 426 | 218 | 224 | 302 | 302 | 212 | 214 | 268 | 268 |
| **CPRJ** | 2899 | M | blood | 0 | 0 | 0 | 0 | 0 | 0 | 0 | 0 | 186 | 190 | 230 | 230 | 420 | 420 | 0 | 0 | 0 | 0 | 0 | 0 | 268 | 272 |
| **FPZSP** | 2699 | M | hair | 218 | 218 | 324 | 328 | 400 | 400 | 274 | 278 | 190 | 190 | 0 | 0 | 420 | 426 | 0 | 0 | 0 | 0 | 0 | 0 | 266 | 270 |
| **FPZSP** | 2435 | M | hair | 210 | 214 | 324 | 332 | 400 | 400 | 278 | 278 | 180 | 180 | 230 | 234 | 420 | 420 | 224 | 224 | 298 | 298 | 212 | 214 | 270 | 270 |
| **FPZSP** | 1915 | M | hair | 210 | 214 | 328 | 336 | 400 | 400 | 282 | 286 | 180 | 180 | 0 | 0 | 420 | 420 | 220 | 220 | 298 | 302 | 212 | 214 | 270 | 274* |
| **FPZSP** | 2309 | M | hair | 214 | 218 | 328 | 340 | 396 | 400 | 274 | 286 | 180 | 190 | 0 | 0 | 420 | 420 | 224 | 224 | 298 | 298 | 210 | 214 | 270 | 270 |
| **FPZSP** | 2331 | M | hair | 0 | 0 | 0 | 0 | 0 | 0 | 0 | 0 | 190 | 190 | 230 | 238* | 424* | 426 | 218 | 220 | 298 | 302 | 210 | 212 | 270 | 272* |
| **FPZSP** | 1954 | M | hair | 0 | 0 | 0 | 0 | 0 | 0 | 0 | 0 | 180 | 180 | 0 | 0 | 420 | 420 | 220 | 224 | 298 | 302 | 212 | 214 | 270 | 270 |
| **FPZSP** | 1850 | M | hair | 210 | 214 | 316 | 332 | 400 | 400 | 278 | 282 | 180 | 180 | 230 | 230 | 0 | 0 | 220 | 222* | 0 | 0 | 212 | 212 | 258* | 266 |
| **FPZSP** | 1408 | F | hair | 210 | 218 | 328 | 328 | 400 | 400 | 274 | 286 | 180 | 180 | 230 | 236 | 420 | 420 | 224 | 224 | 298 | 302 | 210 | 212 | 270 | 270 |
| **FPZSP** | 1657 | M | hair | 214 | 218 | 0 | 0 | 0 | 0 | 0 | 0 | 180 | 190 | 0 | 0 | 420 | 420 | 220 | 224 | 298 | 298 | 212 | 214 | 270 | 270 |
| **FPZSP** | 1803 | M | hair | 210 | 210 | 328 | 336 | 396 | 400 | 274 | 286 | 180 | 180 | 230 | 236 | 420 | 420 | 220 | 224 | 0 | 0 | 210 | 210 | 266 | 270 |
| **FPZSP** | 1825 | F | hair | 210 | 210 | 316 | 336 | 400 | 404* | 282 | 286 | 180 | 190 | 234 | 236 | 420 | 422 | 220 | 220 | 298 | 302 | 210 | 214 | 266 | 270 |
| **FPZSP** | 2093 | F | hair | 210 | 214 | 328 | 340 | 400 | 400 | 274 | 274 | 180 | 190 | 228 | 228 | 420 | 420 | 224 | 224 | 298 | 298 | 210 | 212 | 270 | 270 |
| **FPZSP** | 2385 | M | hair | 210 | 214 | 316 | 316 | 400 | 408 | 278 | 298 | 190 | 190 | 0 | 0 | 420 | 424* | 218 | 220 | 0 | 0 | 210 | 210 | 270 | 270 |
| **FPZSP** | 2539 | M | hair | 214 | 214 | 332 | 332 | 396 | 400 | 282 | 298 | 190 | 190 | 0 | 0 | 420 | 420 | 0 | 0 | 298 | 298 | 210 | 210 | 266 | 270 |
| **FPZSP** | 2550 | F | hair | 214 | 218 | 324 | 332 | 400 | 400 | 278 | 278 | 180 | 190 | 0 | 0 | 420 | 420 | 224 | 224 | 298 | 298 | 210 | 212 | 270 | 270 |
| **FPZSP** | 2551 | F | hair | 210 | 214 | 336 | 340 | 400 | 400 | 286 | 286 | 190 | 190 | 228 | 234 | 420 | 420 | 224 | 224 | 298 | 298 | 212 | 212 | 270 | 270 |
| **FPZSP** | 2639 | F | hair | 214 | 218 | 328 | 332 | 400 | 400 | 278 | 286 | 190 | 190 | 0 | 0 | 420 | 424* | 218 | 224 | 298 | 298 | 210 | 212 | 266 | 272* |
| **FPZSP** | 2640 | M | hair | 210 | 210 | 316 | 328 | 400 | 404* | 282 | 286 | 180 | 190 | 234 | 236 | 420 | 420 | 220 | 224 | 298 | 298 | 210 | 214 | 266 | 270 |
| **FPZSP** | 2641 | F | hair | 210 | 210 | 336 | 340 | 400 | 400 | 286 | 286 | 180 | 180 | 0 | 0 | 420 | 420 | 220 | 224 | 0 | 0 | 210 | 214 | 270 | 270 |
| **FPZSP** | 2694 | M | hair | 210 | 218 | 324 | 332 | 400 | 400 | 278 | 286 | 190 | 190 | 0 | 0 | 420 | 422 | 220 | 224 | 298 | 302 | 210 | 214 | 270 | 270 |
| **FPZSP** | 2704 | M | hair | 214 | 218 | 316 | 324 | 400 | 400 | 278 | 286 | 190 | 190 | 0 | 0 | 420 | 420 | 220 | 224 | 298 | 298 | 210 | 210 | 266 | 270 |
| **FPZSP** | 2705 | M | hair | 210 | 218 | 328 | 340 | 400 | 412* | 286 | 286 | 180 | 180 | 230 | 236 | 420 | 420 | 220 | 224 | 298 | 298 | 210 | 210 | 270 | 270 |
| **FPZSP** | 2760 | M | hair | 218 | 218 | 324 | 332 | 400 | 400 | 278 | 278 | 180 | 190 | 234 | 236 | 420 | 420 | 220 | 224 | 298 | 298 | 212 | 214 | 270 | 270 |
| **FPZSP** | 2761 | F | hair | 210 | 214 | 316 | 340 | 400 | 400 | 282 | 286 | 180 | 190 | 230 | 234 | 420 | 420 | 220 | 220 | 298 | 298 | 210 | 214 | 270 | 270 |
| **FPZSP** | 2774 | F | hair | 210 | 210 | 316 | 336 | 400 | 400 | 282 | 286 | 180 | 190 | 236 | 236 | 420 | 422 | 220 | 220 | 298 | 298 | 210 | 210 | 266 | 270 |
| **FPZSP** | 2848 | M | hair | 210 | 210 | 328 | 332 | 396 | 400 | 262 | 286 | 180 | 190 | 0 | 0 | 420 | 422 | 220 | 224 | 298 | 302 | 210 | 214 | 266 | 270 |
| **FPZSP** | 2384 | F | hair | 210 | 214 | 328 | 340 | 400 | 400 | 262 | 286 | 190 | 190 | 0 | 0 | 0 | 0 | 220 | 220 | 298 | 302 | 210 | 210 | 0 | 0 |
| **FPZSP** | 2697 | F | hair | 210 | 218 | 328 | 340 | 396 | 396 | 262 | 286 | 180 | 190 | 0 | 0 | 420 | 420 | 220 | 222* | 298 | 298 | 210 | 210 | 266 | 270 |
| **FPZSP** | 2564 | F | hair | 210 | 214 | 336 | 340 | 400 | 416* | 262 | 262 | 180 | 190 | 0 | 0 | 420 | 426 | 218 | 224 | 298 | 302 | 210 | 210 | 0 | 0 |
| **FPZSP** | 2385 | M | hair | 218 | 218 | 328 | 332 | 400 | 400 | 278 | 286 | 180 | 190 | 0 | 0 | 420 | 424* | 218 | 220 | 298 | 302 | 210 | 210 | 270 | 270 |
| **FPZSP** | 2079 | F | hair | 214 | 218 | 328 | 328 | 400 | 400 | 274 | 286 | 180 | 190 | 230 | 230 | 420 | 420 | 224 | 224 | 298 | 298 | 210 | 214 | 270 | 270 |
| **FPZSP** | 2109 | F | hair | 210 | 218 | 332 | 340 | 396 | 400 | 286 | 286 | 180 | 190 | 230 | 230 | 420 | 420 | 224 | 224 | 298 | 298 | 212 | 214 | 270 | 270 |
| **FPZSP** | 2245 | F | hair | 218 | 218 | 332 | 332 | 396 | 400 | 286 | 286 | 180 | 180 | 0 | 0 | 420 | 420 | 220 | 224 | 298 | 302 | 210 | 214 | 0 | 0 |
| **FPZSP** | 2273 | M | hair | 214 | 214 | 332 | 332 | 400 | 400 | 282 | 298 | 180 | 180 | 230 | 234 | 420 | 420 | 0 | 0 | 298 | 302 | 210 | 214 | 270 | 270 |
| **FPZSP** | 2289 | M | hair | 210 | 210 | 328 | 336 | 400 | 400 | 282 | 286 | 0 | 0 | 0 | 0 | 0 | 0 | 0 | 0 | 0 | 0 | 0 | 0 | 0 | 0 |
| **FPZSP** | 2339 | M | hair | 218 | 218 | 324 | 328 | 400 | 400 | 278 | 286 | 180 | 180 | 0 | 0 | 420 | 420 | 220 | 224 | 298 | 298 | 210 | 214 | 0 | 0 |
| **FPZSP** | 2340 | F | hair | 218 | 218 | 324 | 328 | 400 | 400 | 278 | 286 | 180 | 180 | 230 | 236 | 420 | 420 | 220 | 224 | 298 | 302 | 212 | 214 | 270 | 270 |
| **FPZSP** | 2372 | F | hair | 210 | 210 | 336 | 340 | 400 | 400 | 282 | 286 | 180 | 180 | 230 | 236 | 420 | 420 | 220 | 224 | 298 | 298 | 210 | 210 | 266 | 270 |
| **FPZSP** | 2373 | M | hair | 210 | 210 | 316 | 328 | 400 | 404* | 282 | 286 | 180 | 180 | 230 | 236 | 420 | 420 | 220 | 224 | 298 | 302 | 210 | 214 | 266 | 270 |
| **FPZSP** | 2533 | M | hair | 210 | 210 | 316 | 336 | 396 | 400 | 274 | 274 | 180 | 190 | 230 | 236 | 420 | 426 | 218 | 220 | 298 | 302 | 210 | 210 | 270 | 270 |
| **FPZSP** | 2538 | M | hair | 210 | 214 | 316 | 336 | 396 | 412* | 262 | 278 | 190 | 190 | 234 | 236 | 420 | 420 | 220 | 224 | 298 | 298 | 210 | 214 | 266 | 266 |
| **FPZSP** | 2563 | M | hair | 210 | 214 | 316 | 336 | 400 | 412* | 274 | 278 | 180 | 190 | 234 | 236 | 420 | 420 | 220 | 224 | 298 | 298 | 210 | 214 | 270 | 270 |
| **FPZSP** | 2645 | F | hair | 210 | 214 | 336 | 340 | 400 | 400 | 286 | 286 | 180 | 190 | 234 | 236 | 420 | 420 | 220 | 220 | 298 | 298 | 210 | 210 | 270 | 270 |
| **FPZSP** | 2703 | F | hair | 210 | 210 | 316 | 336 | 400 | 404* | 282 | 286 | 190 | 190 | 230 | 230 | 420 | 420 | 222* | 224 | 298 | 302 | 212 | 212 | 258* | 270 |
| **FPZSP** | 2710 | M | hair | 214 | 214 | 316 | 316 | 400 | 408 | 278 | 298 | 190 | 190 | 234 | 236 | 420 | 422 | 220 | 224 | 0 | 0 | 0 | 0 | 270 | 272* |
| **FPZSP** | 2722 | F | hair | 214 | 214 | 316 | 332 | 396 | 408 | 278 | 298 | 190 | 190 | 230 | 236 | 420 | 426 | 220 | 220 | 298 | 298 | 210 | 212 | 266 | 270 |
| **FPZSP** | 2723 | F | hair | 210 | 214 | 316 | 332 | 396 | 408 | 278 | 298 | 180 | 190 | 236 | 236 | 420 | 422 | 220 | 220 | 298 | 302 | 210 | 214 | 266 | 270 |
| **FPZSP** | 2846 | M | hair | 210 | 210 | 316 | 316 | 400 | 408 | 298 | 298 | 180 | 180 | 234 | 236 | 420 | 420 | 220 | 220 | 298 | 302 | 210 | 214 | 266 | 270 |
| **FPZSP** | 2717 | M | hair | 210 | 210 | 316 | 316 | 396 | 408 | 298 | 298 | 180 | 190 | 236 | 236 | 420 | 420 | 220 | 220 | 0 | 0 | 0 | 0 | 266 | 270 |
| **NA (%)** |  |  |  | **5.8** | | **5.8** | | **8.6** | | **1.0** | | **1.9** | | **27.9** | | **4.8** | | **5.8** | | **1.6** | | **8.6** | | **3.8** | |
