# Supplementary material for: Genetic diversity in ex situ populations of the endangered Leontopithecus chrysomelas and implications for its conservation
Source: PLoS One. 2023 Aug 2;18(8):e0288097. doi: 10.1371/journal.pone.0288097 (PMC10395972; doi:10.1371/journal.pone.0288097)
Supplement: S2 Table — K: number of genetic clusters. (DOCX) [file pone.0288097.s002.docx]

**S2 Table.** Values of Ln’(K) and Delta K for the Structure analysis measured with Structure Harvester for the Brazilian captive *Leontopithecus chrysomelas* populations using a panel of 11 microsatellite loci (Lchu1, Lchu3, Lchu4, Lchu5, Lchu6, Lchu8, Lchu9, Leon2, Leon21, Leon27, Leon30). K: genetic cluster number.

| **K** | **Repeats** | **Mean LnP(K)** | **Stdev LnP(K)** | **Ln'(K)** | **\|Ln''(K)\|** | **Delta K** |
| --- | --- | --- | --- | --- | --- | --- |
| 1 | 6 | -2624.1500 | 0.7368 | - | - | - |
| 2 | 6 | -2500.3000 | 78.8820 | 123.8500 | 1536.6333 | 19.4801 |
| 3 | 6 | -3913.0833 | 1094.6646 | -1412.7833 | 1085.8166 | 0.99192 |
| 4 | 6 | -6411.6833 | 626.5671 | -2498.6000 | 4119.1666 | 6.57418 |
| 5 | 6 | -13029.4500 | 6424.0829 | -6617.7666 | 7617.8333 | 1.18582 |
| 6 | 6 | -12029.3833 | 2747.4779 | 1000.0667 | 4352.3500 | 1.58413 |
| 7 | 6 | -15381.6667 | 4680.6395 | -3352.3283 | 2098.3500 | 0.44834 |
