# Supplementary material for: Genetic diversity in ex situ populations of the endangered Leontopithecus chrysomelas and implications for its conservation
Source: PLoS One. 2023 Aug 2;18(8):e0288097. doi: 10.1371/journal.pone.0288097 (PMC10395972; doi:10.1371/journal.pone.0288097)
Supplement: S3 Table — K: number of genetic clusters. HWE: Hardy Weinberg Equilibrium. (DOCX) [file pone.0288097.s003.docx]

**S3 Table.** Values of Ln’(K) and Delta K for the Structure analysis measured with Structure Harvester for the Brazilian captive *Leontopithecus chrysomelas* populations using only the eight microsatellite loci with no HWE deviations (Lchu1, Lchu3, Lchu4, Lchu6, Lchu8, Leon2, Leon21, Leon27). K: genetic cluster number. HWE: Hardy Weinberg Equilibrium.

| **K** | **Repeats** | **Mean LnP(K)** | **Stdev LnP(K)** | **Ln'(K)** | **\|Ln''(K)\|** | **Delta K** |
| --- | --- | --- | --- | --- | --- | --- |
| 1 | 5 | -1797.3200 | 0.7225 | - | - | - |
| 2 | 5 | -1686.5200 | 0.6979 | 110.800000 | 41.300000 | 59.181446 |
| 3 | 5 | -1617.0200 | 1.3387 | 69.500000 | 78.480000 | 58.625963 |
| 4 | 5 | -1626.0000 | 23.7564 | -8.980000 | 52.000000 | 2.188887 |
| 5 | 5 | -1686.9800 | 83.5561 | -60.980000 | 1.460000 | 0.017473 |
| 6 | 5 | -1749.4200 | 30.6237 | -62.440000 | - | - |
