# Supplementary material for: Genetic diversity in ex situ populations of the endangered Leontopithecus chrysomelas and implications for its conservation
Source: PLoS One. 2023 Aug 2;18(8):e0288097. doi: 10.1371/journal.pone.0288097 (PMC10395972; doi:10.1371/journal.pone.0288097)
Supplement: S4 Table — CPRJ: Primatology Center of Rio de Janeiro; FPZSP: Zoological Park Foundation of São Paulo. (DOCX) [file pone.0288097.s004.docx]

**S4 Table.** Summary of the effective population size (Ne) and confidence interval (95% CI), measured with NeEstimator 2.0 software, for the Brazilian captive populations of *Leontopithecus chrysomelas*. CPRJ: Primatology Center of Rio de Janeiro; FPZSP: Zoological Park Foundation of São Paulo.

|  | **CPRJ** | | | | **FPZSP** | | | |
| --- | --- | --- | --- | --- | --- | --- | --- | --- |
| **Lowest Allele Frequency Used** | 0.05 | 0.02 | 0.01 | 0+ | 0.05 | 0.02 | 0.01 | 0+ |
|  | **Linkage disequilibrium Method** | | | | | | | |
| **Estimated Ne^ =** | 23 | 24.5 | 25.8 | 26.9 | 8.6 | 11.2 | 11.3 | 11.3 |
|  | **95% CIs for Ne^** | | | | | | | |
| **Parametric** | 16.9 | 18.4 | 19.4 | 20.1 | 5.3 | 7.9 | 8 | 8 |
|  | 32.2 | 33.5 | 35.5 | 37.3 | 12.6 | 15.7 | 15.7 | 15.7 |
| **JackKnife on Samples** | 13.1 | 14.6 | 16.6 | 16.9 | 3.4 | 6.3 | 6.4 | 6.4 |
|  | 44.8 | 45.3 | 43.4 | 47.3 | 18.1 | 18.9 | 18.9 | 18.9 |
