# Supplementary material for: Genetic diversity in ex situ populations of the endangered Leontopithecus chrysomelas and implications for its conservation
Source: PLoS One. 2023 Aug 2;18(8):e0288097. doi: 10.1371/journal.pone.0288097 (PMC10395972; doi:10.1371/journal.pone.0288097)
Supplement: S5 Table — Ne: effective population sizes, AN: number of alleles, HO: observed heterozygosity and HE: expected heterozygosity. (DOCX) [file pone.0288097.s005.docx]

**S5 Table.** Comparison of mean genetic diversity values for the Brazilian captive *Leontopithecus chrysomelas* populations retained over 100 years for the simulations of 0%, 20% and 50% of bottleneck for the Zoological Park Foundation of São Paulo (FPZSP), the Primatology Center of Rio de Janeiro (CPRJ) and the FPZSP-CPRJ. Ne: effective population sizes, A_N_: number of alleles, H_O_: observed heterozygosity and H_E_: expected heterozygosity.

| **CURRENT ESTIMATED GENETIC DIVERSITY** | | | | |
| --- | --- | --- | --- | --- |
| **Populations** | **Ne** | **A_N_** | **H_O_** | **H_E_** |
| **FPZSP** | **11** | 4.18 | 0.55 | 0.56 |
| **CPRJ** | **26** | 5.09 | 0.65 | 0.64 |
| **FPZSP-CPRJ** | **20** | 5.36 | 0.61 | 0.64 |
| **PREDICTED GENETIC DIVERSITY AFTER 100 YEARS** | | | | |
|  | **0% Bottleneck** |  | | |
| **Populations** | **Ne** | **A_N_** | **H_O_** | **H_E_** |
| **FPZSP** | 11 | 1.84 | 0.27 | 0.26 |
| **CPRJ** | 26 | 2.70 | 0.47 | 0.45 |
| **FPZSP-CPRJ** | 20 | 5.02 | 0.41 | 0.41 |
|  | **20% Bottleneck** |  |  |  |
| **Populations** | **Ne** | **A_N_** | **H_O_** | **H_E_** |
| **FPZSP** | 8 | 1.62 | 0.22 | 0.21 |
| **CPRJ** | 20 | 2.40 | 0.43 | 0.41 |
| **FPZSP-CPRJ** | 16 | 4.80 | 0.37 | 0.37 |
|  | **50% Bottleneck** |  |  |  |
| **Populations** | **Ne** | **A_N_** | **H_O_** | **H_E_** |
| **FPZSP** | 6 | 0.00 | 0.00 | 0.00 |
| **CPRJ** | 13 | 1.94 | 0.35 | 0.32 |
| **FPZSP-CPRJ** | 10 | 4.26 | 0.29 | 0.28 |
