# Supplementary material for: Genetic diversity in ex situ populations of the endangered Leontopithecus chrysomelas and implications for its conservation
Source: PLoS One. 2023 Aug 2;18(8):e0288097. doi: 10.1371/journal.pone.0288097 (PMC10395972; doi:10.1371/journal.pone.0288097)
Supplement: S6 Table — CPRJ: Primatology Center of Rio de Janeiro; FPZSP: Zoological Park Foundation of São Paulo. AR: alleles richness, HE: expected heterozygosity, HO: observed heterozygosity, FIS: inbreeding coefficient. HWE: Hardy Weinberg Equilibrium. * Significant statistically differences P < 0.05. (DOCX) [file pone.0288097.s006.docx]

**S6 Table.** Summary of the P values for significant differences between the genetic diversity parameters (GD) for the Brazilian captive populations of *Leontopithecus chrysomelas*, using the total panel of 11 microsatellite loci (Lchu1, Lchu3, Lchu4, Lchu5, Lchu6, Lchu8, Lchu9, Leon2, Leon21, Leon27 and Leon30) and the reduced panel of eight microsatellite loci (Lchu1, Lchu3, Lchu4, Lchu6, Lchu8, Leon2, Leon21and Leon27), after exclusion of loci with HWE deviation. CPRJ: Primatology Center of Rio de Janeiro; FPZSP: Zoological Park Foundation of São Paulo. A_R_: alleles richness, H_E_: expected heterozygosity, H_O_: observed heterozygosity, F_IS_: inbreeding coefficient. HWE: Hardy Weinberg Equilibrium. * Significant statistically differences P < 0.05.

| ***P values*** | | | | | |
| --- | --- | --- | --- | --- | --- |
| **GD** | **Shapiro** (11 loci) | | **Shapiro** (8 loci) | **Levene** | ***t* teste** |
| **CPRJ** | | | | | |
| **A_R_** | 0.79 | 0.41 | | 0.94 | 0.65 |
| **H_E_** | 0.28 | 0.73 | | 0.75 | 0.47 |
| **H_O_** | 0.79 | 0.72 | | 0.93 | 0.90 |
| **F_IS_** | 0.46 | 0.22 | | 0.64 | 0.53 |
| **FPZSP** | | | | | |
| **A_R_** | 0.35 | | 0.36 | 0.74 | 0.70 |
| **H_E_** | 0.93 | | 0.99 | 0.48 | 0.76 |
| **H_O_** | 0.6 | | 0.47 | 0.47 | 0.92 |
| **F_IS_** | 0.61 | | 0.56 | 0.85 | 0.95 |
