# Supplementary material for: Genetic diversity in ex situ populations of the endangered Leontopithecus chrysomelas and implications for its conservation
Source: PLoS One. 2023 Aug 2;18(8):e0288097. doi: 10.1371/journal.pone.0288097 (PMC10395972; doi:10.1371/journal.pone.0288097)
Supplement: S1 Fig — CPRJ: Primatology Center of Rio de Janeiro; FPZSP: Zoological Park Foundation of São Paulo. (DOCX) [file pone.0288097.s007.docx]

**S1 Fig.** Illustrative map showing (in red) the locations in Brazil where the *ex situ* populations of *Leontopithecus chrysomelas* are under human care and the respective institution names; and (in blue) the locations of the in situ populations that have already been studied by microsatellite data. CPRJ: Primatology Center of Rio de Janeiro; FPZSP: Zoological Park Foundation of São Paulo.
