# Supplementary material for: Genetic diversity in ex situ populations of the endangered Leontopithecus chrysomelas and implications for its conservation
Source: PLoS One. 2023 Aug 2;18(8):e0288097. doi: 10.1371/journal.pone.0288097 (PMC10395972; doi:10.1371/journal.pone.0288097)

**S2 Fig.** Graphical showing the values of DeltaK = mean(|L''(K)|) / sd(L(K)) obtained for the Structure analysis measured with Structure Harvester for the Brazilian captive populations of *Leontopithecus chrysomelas*, indicating the values of genetic clusters (K). CPRJ: Primatology Center of Rio de Janeiro; FPZSP: Zoological Park Foundation of São Paulo.


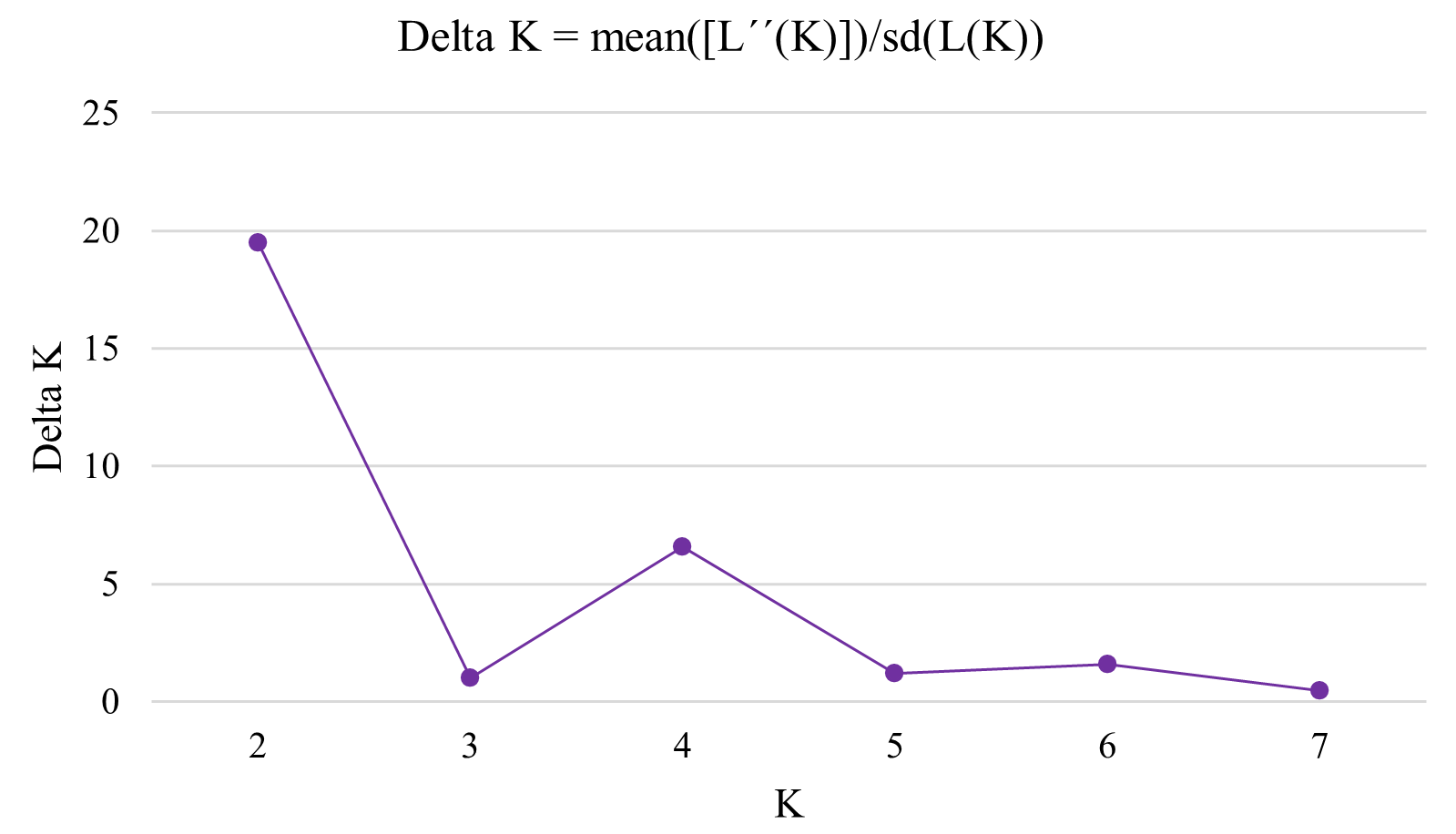

Supplement: S2 Fig — CPRJ: Primatology Center of Rio de Janeiro; FPZSP: Zoological Park Foundation of São Paulo. (DOCX) [file pone.0288097.s008.docx]
