# Supplementary material for: Genetic diversity in ex situ populations of the endangered Leontopithecus chrysomelas and implications for its conservation
Source: PLoS One. 2023 Aug 2;18(8):e0288097. doi: 10.1371/journal.pone.0288097 (PMC10395972; doi:10.1371/journal.pone.0288097)

**S3 Fig.** Structure analysis results for 104 individuals of *Leontopithecus chrysomelas* from the Primatology Center of Rio de Janeiro (CPRJ) and the Zoological Park Foundation of São Paulo (FPZSP), using only the eight microsatellite loci in HWE (Lchu1, Lchu3, Lchu4, Lchu6, Lchu8, Leon2, Leon21and Leon27), considering the most probable K value (K=2). HWE: Hardy Weinberg Equilibrium.


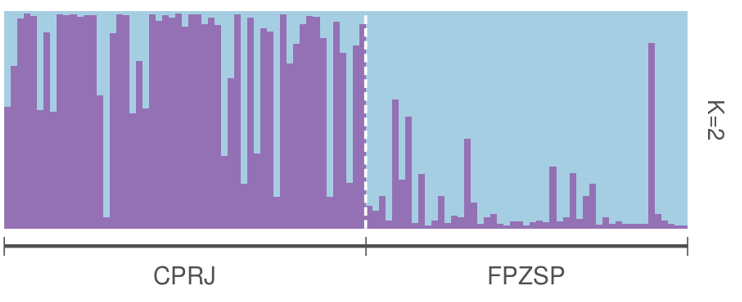

Supplement: S3 Fig — HWE: Hardy Weinberg Equilibrium. (DOCX) [file pone.0288097.s009.docx]
